# A survey of the clinicopathological and molecular characteristics of patients with suspected Lynch syndrome in Latin America

**DOI:** 10.1186/s12885-017-3599-4

**Published:** 2017-09-05

**Authors:** Benedito Mauro Rossi, Edenir Inêz Palmero, Francisco López-Kostner, Carlos Sarroca, Carlos Alberto Vaccaro, Florencia Spirandelli, Patricia Ashton-Prolla, Yenni Rodriguez, Henrique de Campos Reis Galvão, Rui Manuel Reis, André Escremim de Paula, Luis Gustavo Capochin Romagnolo, Karin Alvarez, Adriana Della Valle, Florencia Neffa, Pablo German Kalfayan, Enrique Spirandelli, Sergio Chialina, Melva Gutiérrez Angulo, Maria del Carmen Castro-Mujica, Julio Sanchez de Monte, Richard Quispe, Sabrina Daniela da Silva, Norma Teresa Rossi, Claudia Barletta-Carrillo, Susana Revollo, Ximena Taborga, L. Lena Morillas, Hélène Tubeuf, Erika Maria Monteiro-Santos, Tamara Alejandra Piñero, Constantino Dominguez-Barrera, Patrik Wernhoff, Alexandra Martins, Eivind Hovig, Pål Møller, Mev Dominguez-Valentin

**Affiliations:** 1 Hospital Sirio Libanes, Sao Paulo, Brazil; 2 Molecular Oncology Research Center, Barretos Cancer Hospital, Barretos, SP Brazil; 3 Laboratorio de Oncología y Genética Molecular, Clínica Los Condes, Santiago, Chile; 4 Hospital Fuerzas Armadas, Grupo Colaborativo Uruguayo, Investigación de Afecciones Oncológicas Hereditarias (GCU), Montevideo, Uruguay; 5 Hereditary Cancer Program (PROCANHE), Hospital Italiano, Buenos Aires, Argentina; 6 Servicio de Coloproctologia y Asesoria Genetica en Cancer, Hospital Español de Rosario, Rosario, Argentina; 7 Departamento de Genética da Universidade Federal do Rio Grande do Sul (UFRGS) e Serviço de Genética Médica do Hospital de Clinicas de Porto Alegre (HCPA) & Rede Brasileira de Câncer Hereditário, Porto Alegre, Rio Grande Do Sul Brazil; 8 Clinica del Country, Bogota, Colombia; 9 Oncogenetics Department, Barretos Cancer Hospital, Barretos, SP Brazil; 10 Molecular Oncology Research Center, Barretos Cancer Hospital & Life and Health Sciences Research Institute (ICVS), Health Sciences School, University of Minho, Braga, Portugal; 11 ICVS/3B’s-PT Government Associate Laboratory, Braga, Guimarães Portugal; 12 Centro Universitario de los Altos, Universidad de Guadalajara, Jalisco, Mexico; 13 Equipo Funcional de Genética y Biologia Molecular, Instituto Nacional de Enfermedades Neoplásicas, Lima, Peru; 14 Instituto Nacional de Cancerologia de México, México City, Mexico; 15 Laboratorio de Genética Molecular del Instituto de Servicios de Laboratorio de Diagnóstico e Investigación en Salud (SELADIS), La Paz, Bolivia; 16 Lady Davis Institute for Medical Research and Segal Cancer Center, Jewish General Hospital, Montreal, Quebec, Canada; 17 Department of Otolaryngology-Head and Neck Surgery, McGill University, Montreal, Quebec, Canada; 18 Hospital Privado Universitario de Cordoba, Cordoba, Argentina; 19 Centro de Enfermedades Neoplasicas ONCOVIDA, La Paz, Bolivia; 20 Inserm-U1079-IRIB, UNIROUEN, Normandie Univ, Normandy Centre for Genomic and Personalized Medicine, Rouen, France; 21 Interactive Biosoftware, Rouen, France; 22 Instituto de Ciencias Basicas y Medicina Experimental (ICBME), Hospital Italiano, Buenos Aires, Argentina; 23 Department of Preventive Medicine, Faculty of Medicine, Universidad Nacional Mayor de San Marcos (UNMSM), Lima, Peru; 24 Department of Clinical Molecular Biology (EpiGen), Akershus University Hospital, Lørenskog, Norway; 25 Department of Tumor Biology, Institute for Cancer Research, Oslo University Hospital, Oslo, Norway; 26 Institute of Cancer Genetics and Informatics, Oslo University Hospital, Oslo, Norway; 27 Department of Medical Genetics, Oslo University Hospital, Oslo, Norway; 28 Department of Human Medicine, Universität Witten/Herdecke, Witten, Germany

**Keywords:** Lynch syndrome, Mmr, Latin America, Variants

## Abstract

**Background:**

Genetic counselling and testing for Lynch syndrome (LS) have recently been introduced in several Latin America countries. We aimed to characterize the clinical, molecular and mismatch repair (MMR) variants spectrum of patients with suspected LS in Latin America**.**

**Methods:**

Eleven LS hereditary cancer registries and 34 published LS databases were used to identify unrelated families that fulfilled the Amsterdam II (AMSII) criteria and/or the Bethesda guidelines or suggestive of a dominant colorectal (CRC) inheritance syndrome.

**Results:**

We performed a thorough investigation of 15 countries and identified 6 countries where germline genetic testing for LS is available and 3 countries where tumor testing is used in the LS diagnosis. The spectrum of pathogenic MMR variants included *MLH1* up to 54%, *MSH2* up to 43%, *MSH6* up to 10%, *PMS2* up to 3% and *EPCAM* up to 0.8%. The Latin America MMR spectrum is broad with a total of 220 different variants which 80% were private and 20% were recurrent. Frequent regions included exons 11 of *MLH1* (15%), exon 3 and 7 of *MSH2* (17 and 15%, respectively), exon 4 of *MSH6* (65%), exons 11 and 13 of *PMS2* (31% and 23%, respectively). Sixteen international founder variants in *MLH1*, *MSH2* and *MSH6* were identified and 41 (19%) variants have not previously been reported, thus representing novel genetic variants in the MMR genes. The AMSII criteria was the most used clinical criteria to identify pathogenic MMR carriers although microsatellite instability, immunohistochemistry and family history are still the primary methods in several countries where no genetic testing for LS is available yet.

**Conclusion:**

The Latin America LS pathogenic MMR variants spectrum included new variants, frequently altered genetic regions and potential founder effects, emphasizing the relevance implementing Lynch syndrome genetic testing and counseling in all of Latin America countries.

## Background

LS is caused by a defective mismatch repair (MMR) system, due to the presence of germline defects in at least one of the MMR genes, *MLH1, MSH2, MSH6, PMS2,* or to deletions of the 3′ portion of the *EPCAM* gene [1]. Such variants are here referred to as path_MMR and, when specifying one of the genes, as *path_MLH1, path_MSH2, path_MSH6*, *path_PMS2* or *path_EPCAM* [2, 3]. LS is clinically classified according to the Amsterdam (AMS) criteria and/or the Bethesda guidelines, both relying in clinical information and family history. The Bethesda guidelines also takes into account the microsatellite instability (MSI) tumor marker, which is a signature characteristic of MMR-deficient tumors [4–7]. MSI or immuno-histochemical (IHC) testing of tumors are strategies to select patients for subsequent germline diagnostic testing in blood [8].

LS patients have an increased lifetime risk of colorectal cancer (CRC) (70–80%), endometrial cancer (50–60%), stomach cancer (13–19%), ovarian cancer (9–14%), cancer of the small intestine, the biliary tract, brain as well as carcinoma of the ureters and renal pelvis [9]. The cumulative incidence of any cancer at 70 years of age is 72% for *path_MLH1* and *path_MSH2* carriers but lower in *path_MSH6* (52%) and *path_PMS2* (18%) carriers. *Path_MSH*6 and *path_PMS2* carriers do not have an increased risk for cancer before 40 years of age [2, 3]. The identification of LS patients is a goal because an early diagnosis and intensive screening may predict the disease and/or improve the disease prognosis [2].

The path_MMR variant spectrum of LS has been widely studied in CRC patients from North America, Europe, Australia and Asia. In the past decade, significant advances have been made in molecular testing and genetic counseling for LS in several Latin America countries [10–51].

A broad definition of Latin America is that all countries of the Americas south of the United States are included, with Mexico, Cuba, Puerto Rico and all the countries located in South America as well as the Caribbean Islands. Latin America presents with genetically somewhat different populations, where European and African immigrants have a concentration of the Caucasian population in the southern regions of the continent, whereas in the northern region, the population is predominantly Mestizo (a mixture of European and Amerindian) [52].

Among LS patients, the prevalence of *path*_*MLH1* is 42%, *path*_*MSH2* is 33%, *path*_*MSH6* is 18% and *path*_*PMS2* is 8% [53]. However, recent studies in Latin America LS families described the predominance of *path*_*MSH2* (46%- 66%), followed by *path*_*MLH1* (25%–43%), *path*_*MSH6* (7%–8%), *path*_*PMS2* (2%) and *path*_*EPCAM* (2%) [32, 36, 47]. Some Latin America LS variant spectrum included variants that have not previously been reported and potential founder effects which are useful for future development of genetic testing in these populations. It enables the comparison of LS characteristics and MMR variants across genetic ancestry background differences among these populations [12, 20, 23, 26, 32, 36, 40].

The clinical, molecular and MMR variant spectrum of LS has not been fully studied in all Latin America countries. Our study aims to combine both unpublished register data and published data in order to better describe the LS molecular profile and to update the previously described South American path_MMR variant spectrum study [32].

## Methods

**Fig. 1:**
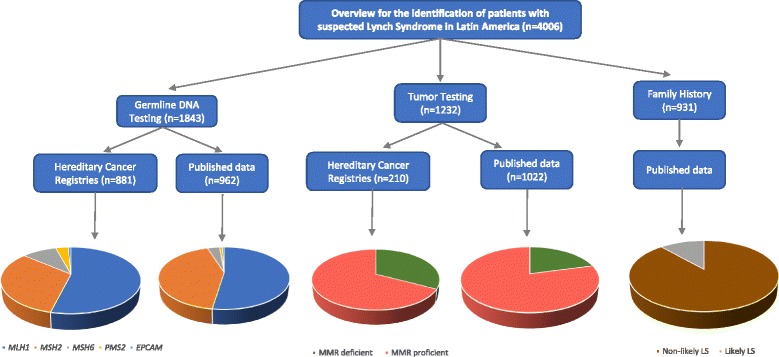
Flowchart depicting the groups of patients with suspected LS in Latin America included in the study. We included unpublished register data and published data from germline MMR testing, tumor testing and family history

Unpublished data from hereditary cancer registries and published data from patients with suspected LS from Latin America have been included in this work. Through research collaborations, data from the Latin America hereditary cancer registers are available following direct contact with the register. The data include results from germline DNA testing, tumor testing (based on MSI analysis and/or IHC) and family history (Fig. 1).

### Hereditary cancer registries

Families that fulfilled the AMSII criteria [4, 5], the Bethesda guidelines [6] and/or other criteria i.e. families suggestive of a dominant CRC inheritance syndrome were selected from 11 hereditary cancer registries from 8 countries: Hospital Italiano (Buenos Aires, Argentina), Hospital Español de Rosario (Rosario, Argentina), Hospital Privado Universitario de Cordoba (Cordoba, Argentina), Centro de Enfermedades Neoplasicas Oncovida (La Paz, Bolivia), Barretos Cancer Hospital (Barretos, Brazil), Hospital de Clinicas de Porto Alegre (Rio Grande do Sul, Brazil), Clinica Las Condes (Santiago, Chile), Clinica del Country (Bogota, Colombia), Instituto Nacional de Cancerologia (Mexico City, Mexico), Instituto Nacional de Enfermedades Neoplasicas (Lima, Peru) and Hospital de las Fuerzas Armadas (Montevideo, Uruguay).

Patients were informed about their inclusion into the registries, which generally contained data on family history, clinical information, age at onset and results of DNA testing or tumor screening in the diagnosis of LS. Written informed consent was obtained from all participants during genetic counseling sessions.

### LS databases

A systematic review was performed in order to identify published reports on MMR variants in LS or hereditary CRC by querying the PubMed, SciELO and Google databases using specific key words (focusing on clinical, tumor or genetic testing information associated with the MMR genes) and taking into account publications in three languages, namely Spanish, English and Portuguese, up to July 2016. The search terms were “Lynch syndrome”, “hereditary colorectal cancer”, “hereditary colorectal cancer and Latin America” and “Lynch syndrome and Latin America”. We also used keywords in association with the names of Latin America countries (e.g., “Lynch syndrome and Colombia”). The results of the search were subsequently screened for the presence of path_MMR variants or tumor screening, clinical diagnosis and family history.

We found 34 LS reports from 12 countries including Argentina [10, 14, 17, 18], Brazil [11, 15, 19, 22, 25, 28, 29, 37, 38, 43], Chile [20, 31], Colombia [12, 16, 23, 48], Mexico [27, 44, 49, 51], El Salvador and Guatemala [51], Paraguay [50], Peru [24, 33, 35, 45], Puerto Rico and Dominican Republic [21, 36], South America [26, 32, 47] and Uruguay [13].

### Germline DNA testing

Genetic testing was generally based on Sanger sequencing of *MLH1*, *MSH2, MSH6* and/or *PMS2* and/or *EPCAM* in 7 participating centers from Argentina (Hospital Italiano de Buenos Aires and Hospital Español de Rosario), Brazil (Barretos Cancer Hospital and Hospital de Clinicas de Porto Alegre), Chile (Clinica Las Condes), Colombia (Clinica del Country) and Uruguay (Hospital de Las Fuerzas Armadas). Multiplex Ligation-dependent Probe Amplification (MLPA) was used to analyze genomic rearrangements in MMR and *EPCAM* genes (SALSA kit P003, MRC-Holland, Amsterdam, Netherland). For *PMS2* analysis, especially for exons 12 to 15, to ensure the correct analysis of *PMS2* and to avoid pseudogene co-amplification, a long-range PCR followed by a nested PCRs strategy was adopted. After amplification, sequencing was performed according to the manufacturer’s instructions.

In addition, we took into consideration the results of germline DNA testing described in 15 previously published LS reports [10, 13, 17, 18, 20, 23, 26, 31, 32, 36, 37, 44, 47, 48, 51].

### Tumor testing

Methods to assess tumor MMR status, e.g. MSI analysis and/or MMR protein staining are being currently used in Cordoba (Argentina), Lima (Peru), La Paz (Bolivia) and Mexico City (Mexico) as an approach to identify potential carriers of germline path_MMR variants. Germline MMR testing is then mandatory to confirm LS cases.

Families from Peru (Instituto Nacional de Enfermedades Neoplasicas) were evaluated for MSI using a 5-mononucleotide marker panel (BAT-25, BAT-26, D2S123, D17S250 and D5S346). Tumors were classified into three categories and defined as MSI high (MSI-H) when ≥2 markers were unstable, MSI low (MSI-L) when one marker was unstable and microsatellite stable (MSS) when none of the markers were unstable. In Bolivia (Centro de Enfermedades Neoplasicas Oncovida), MSI analysis was evaluated by 1-mononucleotide marker panel (BAT-26).

IHC analysis for MMR protein expression was performed on paraffin-embedded tumor tissue sections, as previously described [32]. In Argentina (Hospital Privado Universitario de Cordoba), Mexico (Instituto Nacional de Cancerologia) and Peru, IHC was evaluated using 4-MMR proteins (MLH1, PMS2, MSH2 and MSH6).

Besides the information directly retrieved from these participating centers, we also collected MSI and/or IHC data from 15 LS published reports [14–16, 18, 21, 22, 24, 25, 27, 28, 31, 35, 38, 43, 45].

### Family history

Available data of family history of patients with CRC included 4 published reports from Brazil [19], Mexico [49], Paraguay [50] and Peru [33].

### MMR variants nomenclature and classification

The nomenclature guidelines of the Human Genome Variation Society (HGVS) were used to describe the detected MMR variants [54]. Variants were described by taking into account the following reference sequences: NM_000249.2 (*MLH1*), NM_000251.2 (*MSH2*), NM_000179.2 (*MSH6*), and NM_001322014.1 (*PMS2*). The recurrence or novelty of the identified variants was established by interrogating four databases (in their latest releases as of August 2016): the International Society of Gastrointestinal Hereditary Tumors (InSIGHT) database (accessed via the Leiden Open Variation Database/LOVD), the Universal Mutation Database (UMD), ClinVar, and the Human Gene Mutation Database (HGMD).

The MMR variants were classified according to the 5-tier classification system into the following categories: class 5 (pathogenic), class 4 (likely pathogenic), class 3 (uncertain variants), class 2 (likely not pathogenic) and class 1 (not pathogenic) [55]. Novel MMR variants were considered class 5 if they: a) introduced a premature stop codon in the protein sequence (nonsense or frameshift); b) occurred at the most conserved positions of donor or acceptor splice sites (i.e. IVS ± 1, IVS ± 2); or c) represented whole-exon deletions or duplications.

Well established polymorphisms, Class 1 variants and Class 2 variants were considered normal variants and not included in this study, except for the *MSH6* c.733A > T, which has conflicting interpretations of pathogenicity. We focused on Class 3, Class 4 and Class 5 variants in this study.

In addition, we updated our previous South American LS study [32] according to the 5-tier classification system, with InSiGHT updates [55].

### Splicing-dedicated bioinformatics analysis

The potential impact on RNA splicing induced by the MMR variants was evaluated by focusing on alterations of donor and acceptor splice sites. We took into consideration both the potential impairment of reference splice sites and the possibility of creation of de novo splice sites. The analysis was performed by using the MaxEntScan algorithm [56] interrogated by using the Alamut software (Interactive Biosoftware, France) [57, 58]. For stratification purposes, negative alterations of reference splice sites were deemed important when MaxEntScan scores showed ≥15% decrease relative to corresponding wild-type splice sites [57]. The possibility of variant-induced de novo splice sites was assessed by annotating all increments in local MaxEntScan scores and comparing their values with those of reference splice sites as well as of nearby cryptic splice sites. In this case and for exonic variants, only scores equal or higher to those of the corresponding reference splice site within the same exon (as well as of local cryptic sites) were considered worth noting. In the case of intronic variants, only scores equal or higher to those of the weakest corresponding reference splice site within the same gene (as well as of local cryptic splice sites) were considered as potentially creating de novo splice sites.

### Statistical analysis

Clinical characteristics were described using frequency distributions for categorical variables and summary measures for quantitative variables. To assess comparability of study groups, chi-square test or Fisher’s exact test was used for categorical variables and Student’s t test or Mann-Whitney to compare quantitative variables.

The statistical analyses were performed using the statistical software package IBM SPSS Statistics 20 (SPSS©, Chicago, IL, USA) and STATA 12© (StataCorp. 2011. Stata Statistical Software: Release 12. College Station, TX: StataCorp LP).

## Results

### Path_MMR variants

By combining data provided by 7 participating centers, we identified suspected LS in a total of 881 Latin America individuals belonging to 344 unrelated families (Table 1, Fig. 1). Path_MMR genes were identified in 47% (range 39–64% depending on the participating countries/registries) of the families that fulfilled the AMSII criteria and/or the Bethesda guidelines and/or other criteria (Table 1). When the AMSII criteria were considered, the path_MMR genes detection raised to 64% (91/142), whereas 32% (54/170) and 23% (11/47) fulfilled the Bethesda guidelines and other criteria, respectively. The range of the mean age at diagnosis was 32–45 years for CRC and 43–51 years for endometrial cancer depending on the countries/registries (Table 1). Of the 410 path*_*MMR carriers, *MLH1* was affected in 53.9% (221/410) of the cases*, MSH2* in 32.4% (133/410), *MSH6* in 9.5% (39/410), *PMS2* in 3.4% (14/410) and *EPCAM* in 0.8% (3/410) (Table 1).

**Table 1:** Summary of hereditary cancer registries from Latin America LS families

Latin American Institutions	Number of families	Number of individuals	Families fulfilling^a^			Path_MMR carriers (%)							Path_MMR families fulfilling			Age at CRC diagnosis (mean ± SD)	Age at endometrial cancer diagnosis (mean ± SD)
			AMSII	Revised Bethesda	Other criteria	Path_MMR carriers	Path_MMR non-carriers	*Path_MLH1* carriers	*Path_MSH2* carriers	*Path_MSH6* carriers	*Path_PMS2* carriers	*Path_EPCAM* carriers	AMSII	Revised Bethesda	Other criteria		
Barretos Cancer Hospital (São Paulo, Brazil)	125	369	15	95	30	172 (46.6)	197 (53.4)	79 (45.9)	51 (29.7)	32 (18.6)	10 (5.8)	0	12	48	10	na	na
Clinica Las Condes (Santiago, Chile)	100	212	44	47	9	82 (38.7)	130 (61.3)	63 (76.8)	14 (17.1)	0	2 (2.4)	3 (3.7)	24	4	0	40 (10.5)	48.8 (11.5)
Hospital de las Fuerzas Armadas (Montevideo, Uruguay)	29	177	26	1	2	101 (57.1)	76 (42.9)	55 (54.5)	39 (38.6)	7 (6.9)	0	0	19	0	0	39.9 (9.6)	44.4 (11.9)
Hospital Italiano (Buenos Aires, Argentina)	54	75	35	14	5	26 (34.7)	49 (65.3)	11 (42.3)	15 (57.7)	0	0	0	18	0	0	45.8 (7.01)	43.8 (7.08)
Hospital Español de Rosario (Rosario, Argentina)	13	25	6	7	0	16 (64)	9 (36)	5 (31.3)	10 (62.5)	0	1 (6.2)	0	6	2	0	40.4 (10.4)	51 (na)
Hospital das Clinicas (Porto Alegre, Brazil)	18	18	12	6	0	11(61.1)	7 (38.9)	8 (72.7)	3 (27.3)	0	na	0	11	0	0	42.1 (7.8)	na
Clinica del Country (Bogota, Colombia)	5	5	4	0	1	2 (40)	3 (60)	0	1 (50)	0	1 (50)	0	1	0	1	32 (na)	na
Total	344	881	142	170	47	410 (46.5)	471 (53.5)	221 (53.9)	133 (32.4)	39 (9.5)	14 (3.4)	3 (0.8)	91	54	11		

^a^some families meet more than one clinical criteria; LS: Lynch syndrome; CRC: colorectal cancer; MMR: mismatch repair; SD: standard deviation; na: not applied; Path_MMR: Pathogenic (disease-causing) variant of an MMR gene; path_MLH1: pathogenic variant of the MLH1 gene; path_MSH2: pathogenic variant of the MSH2 gene; path_MSH6: pathogenic variant of the MSH6 gene; path_PMS2: pathogenic variant of the PMS2 gene

Fifteen published data from Argentina, Brazil, Chile, Colombia, Dominican Republic, El Salvador, Guatemala, Mexico, Puerto Rico, South America and Uruguay contained information about 962 tested individuals belonging to 1514 suspected LS families (Table 2, Fig. 1). Path_MMR variants were identified in 40% (389/962) (range 25–100% in the different databases/countries) of the families that fulfilled the AMSII criteria and/or the Bethesda guidelines and/or other criteria. The range of the mean age at diagnosis was 35–45 years for CRC and 41–49 years for endometrial cancer in the different databases (Table 2). Of the 389 path*_*MMR carriers, *MLH1* was affected in 52.4% (204/389)*, MSH2* in 42.7% (166/389), *MSH6* in 3.6% (14/389), *PMS2* in 0.8% (3/389) and *EPCAM* in 0.5% (2/389) (Table 2).

**Table 2:** Summary of published data from Latin America LS families

Latin America LS published databases	Number of families	Number of individuals	Age at CRC diagnosis (mean ± SD)	Age at endometrial cancer diagnosis (mean ± SD)	AMSII	Revised Bethesda	Other criteria	Path_MMR carriers (%)	Path_MMR non- carriers (%)	*Path_MLH1* carriers (%)	*Path_MSH2* carriers (%)	*Path_MSH6* carriers (%)	*Path_PMS2* carriers (%)	*Path_EPCAM* carriers (%)
Mendoza, Argentina [10]	1	17	na	na	1	0	0	9(52.9)	8(47.1)	0	9100)	na	na	na
Sao, Paulo, Brazil [11]	25	25	45.7(na)	na	6	18	1	10(40)	15(60)	8(80)	2(20)	na	na	na
Montevideo, Uruguay [13]	12	12	45	na	12	na	na	3(25)	9(75)	2(67)	1(33)	0	na	na
Bogota, Colombia [12, 48]	23	23	na	na	11	12	na	11(47.8)	12(52.2)	10(91)	1(9)	na	na	na
Buenos Aires, Argentina [17]	43	11	na	na	43	0	na	5(45.5)	6 (54.5)	2(40)	3(60)	na	na	na
Mexico, El Salvador and Guatemala [51]	13	14	38.7(na)	na	5	9	na	11(78.6)	3(21.4)	7(64)	4 (36)	na	na	na
Santiago, Chile [20]	21	20	na	na	14	7	na	9(45)	11(55)	6(30)	3(15)	na	na	na
Antioquia, Colombia [23]	1	20	na	na	1	na	na	7(35)	13(65)	7(100)	0	na	na	na
Southeastern Brazil, Buenos Aires and Montevideo [26]	123	123	na	na	57	66	na	34(27.6)	89(72.4)	20(59)	14(41)	na	na	na
Santiago, Chile [31]	35	35	na	na	19	16	na	21(60)	14(40)	14(67)	5(24)	2(9)	na	na
South America [32]	267	267			147	120	na	99(37.1)	168(62.9)	59(60)	40(40)	na	na	na
*Buenos Aires, Argentina*	28	na	44.3(6.2)	46.3(5.5)										
*Montevideo, Uruguay*	25	na	35.1(7.6)	41.5(8.3)										
*Santiago, Chile*	50	na	35.7(10.7)	41.1(8.8)										
*Barretos, Brazil)*	23	na	39.4(13.8)	49.8(5.3)										
*Colombia*	13	na	na	na										
*Southeastern Brazil*	128	na	42.3(11.4)	48.8(2.4)										
Puerto Rico and Dominican Republic [36]	78	31	44.4(na)	44 (na)	na	na	na	22(71)	9 (29)	8(36)	13(59)	1(5)	na	na
Southeastern Brazil [37]	116	116	42.4(na)	46 (na)	49	67	na	45(38.8)	71(61)	15(33)	25(56)	4(9)	1(2)	na
Jalisco, Mexico [44]	3	5	37.7(na)	na	3	0	na	5(100)	0	4(80)	1(20)	na	na	na
South America [47]	243	243			na	na	na	98(40.3)	145 (56.7)	42(43)	45(46)	7(7)	2(2)	2(2)
*Buenos Aires, Argentina*	48	na	44(na)	45(na)										
*Montevideo, Uruguay*	16	na	42.3(na)	48.8(na)										
*Santiago, Chile*	27	na	41.3(na)	43.6(na)										
*Barretos, Brazil)*	23	na	39.4(na)	49.8(na)										
*Colombia*	13	na	na	na										
*Southeastern Brazil*	116	na	42.4(na)	46(na)										
*Total*	1514	962			368	315	1	389 (40.4)	573 (59.6)	204 (52.4)	166 (42.7)	14 (3.6)	3 (0.8)	2 (0.5)

LS: Lynch syndrome; CRC: colorectal cancer; MMR: mismatch repair; SD: standard deviation; na: not applied; Path_MMR: Pathogenic (disease-causing) variant of an MMR gene; path_MLH1: pathogenic variant of the MLH1 gene; path_MSH2: pathogenic variant of the MSH2 gene; path_MSH6: pathogenic variant of the MSH6 gene; path_PMS2: pathogenic variant of the PMS2 gene

### Latin America MMR variants

**Fig. 2:**
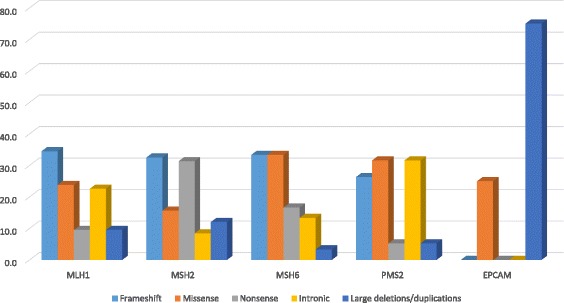
Type of MMR variants in Latin America LS families

In total, 220 unique alterations were identified, including 71 frameshift variants, 50 missense variants, 40 nonsense variants, 36 intronic variants and 23 large deletions/duplications. Frameshift and missense variants were the most common alterations (32% and 23%, respectively), followed by nonsense variants (18%), intronic variants (16%) and large deletions/duplications (11%) (Fig. 2, Table 3).

**Table 3:** Spectrum of MMR variants in Latin America LS families

Gene	cNomenclature	pNomenclature	Exon	Reported/Current Study classification	References	Country	Number of families	RNA splicing-dedicated in silico analysis		
								WT MaxEntScan score	Variant MaxEntScan score	Difference in MaxEntScan score between variant and WT (%)
***MLH1***	c.(?_-198)_116 +?del		1	Class 5	InSIGHT	Chile	2	nd	nd	nd
	c.83C > T	p.Pro28Leu	1	Class 5	InSIGHT	Brazil	2	8.60	8.60	0
	c.91_92delinsTG	p.Ala31Cys	1	Class 3	InSIGHT	Uruguay	1	8.60	8.60	0
	c.116G > A	p.Cys39Tyr	1	Class 4	InSIGHT	Argentina	1	8.60	2.61	−70
	c.117-1G > T		1i	Class 5	HGMD	Brazil	1	7.22	0.00	−100
	c.117-?_207 +?del	p.Cys39^*^	2	Class 5	InSIGHT	Brazil	1	nd	nd	nd
	c.117-691_306 + 1011del	p.Cys39Trpfs^*^6	2–3	Class 5	InSIGHT	Mexico	1	7.22	7.22	0
	c.119delT	p.Leu40^*^	2	Class 5	InSIGHT	Uruguay	1	7.22	7.25	0
	c.122A > G	p.Asp41Gly	2	Class 3	InSIGHT	Brazil	1	7.22	7.22	0
	c.199G > A	p.Gly67Arg	2	Class 5	InSIGHT	Argentina	1	10.45	10.45	0
	c.211G > T	p.Glu71^*^	3	Class 5	InSIGHT	Brazil	1	8.11	8.11	0
	**c.225delT**	p.Ile75Metfs	3	Not reported/Class 5	Current study	Brazil	1	8.11	8.11	0
	c.289 T > G	p.Tyr97Asp	3	Class 3	32	Uruguay	2	9.85	9.85	0
	c.306 + 5G > A		3i	Class 5	UMD, HGMD	Brazil	1	9.85	7.20	−27
	c.307-2A > G		1i	Class 5	UMD (modified from 51)	Guatemala	1	10.90	0.00	−100
	c.332C > T	p.Ala111Val	4	Class 4	InSIGHT	Brazil	1	10.90	10.90	0
	C.336 T > A	p.His112Gln	4	Class 3	32	Argentina	1	10.90	10.90	0
	c.350C > T	p.Thr117Met	4	Class 5	InSIGHT	Uruguay, Argentina	5	8.73	8.73	0
	c.421C > G	p.Pro141Ala	5	Class 3	12	Colombia	1	10.65	10.65	0
	c.454-501_546-1098del	p.Glu153Phefs^*^8	5i	Class 5	InSIGHT	Uruguay	1	6.39	6.39	0
	c.503dupA	p.Asn168Lysfs^*^4	6	Class 5	InSIGHT	Chile	1	8.68	8.68	0
	c.503delA	p.Glu172Asnfs^*^30	6	Class 5	32	Brazil	1	8.68	8.68	0
	c.545 + 3A > G	p.Glu153Valfs^*^9	6i	Class 5	InSIGHT	Brazil	2	8.68	4.95	−43
	c.588 + 2 T > A^b^		7i	Class 4	26	Brazil	1	9.72	0.00	−100
	c.588 + 5G > C		7i	Class 3	InSIGHT	Brazil	1	9.72	4.33	**−55**
	**c.588 + 5G > T**		7i	Not reported	Current study	Argentina	1	9.72	3.64	**−63**
	c.665delA	p.Asn222Metfs^*^7	8	Class 5	InSIGHT	Uruguay	4	9.22	9.22	0
	c.676C > T	p.Arg226^*^	8	Class 5	InSIGHT	Argentina, Mexico	2	9.22	7.12	−23
	c.677G > A^c^	p.Gln197Argfs^*^8	8	Class 5	InSIGHT	Argentina, Brazil	2	9.22	5.00	−46
	c.677 + 1G > A		8i	Class 4	InSIGHT	Brazil	2	9.22	0.00	−100
	c.677 + 5G > A		8i	Class 4	UMD	Chile, Argentina	2	9.22	4.42	−52
	c.779 T > G	p.Leu260Arg	9	Class 5	InSIGHT	Brazil	1	10.43	10.43	0
	c.790 + 1G > A	p.Glu227_Ser295del	9i	Class 5	InSIGHT	Chile, Colombia	3	10.43	0.00	−100
	c.791–4_795delTTAGATCGT		10	Class 5	26	Brazil	2	9.42	0.00	−100
	**c.794G > C** ^**d**^	p.Arg265Pro	10	Not reported	80	Chile	1	9.42	9.42	0
	**c.884 + 5 T > C**		10i	Not reported	Current study	Argentina	1	9.43	10.52	12
	**c.888_890delAGAinsC**	p.Leu296Phefs	11	Not reported/Class 5	Current study	Brazil	1	10.46	10.46	0
	c.901C > T	p.Gln301^*^	11	Class 5	InSIGHT	Chile	1	10.46	10.46	0
	**c.911delA**	p.Asp304Valfs^*^63	11	Not reported/Class 5	Current study	Uruguay	1	10.46	10.46	0
	c.997_1000delAAGC	p.Lys333Serfs^*^33	11	Class 5	78	Chile	1	7.20	7.20	0
	c.1013A > G	p.Asn338Ser	11	Class 3	InSIGHT	Brazil	1	7.20	7.20	0
	c.1023delG	p.Met342Cysfs^*^25	11	Class 5	InSIGHT	Puerto Rico	2	7.20	7.20	0
	c.1038 + 1G > T	p.Thr347PhefsX14	11i	Class 5	31	Chile	1	7.20	0.00	−100
	**c.1039-6delT**		11i	Not reported	Current study	Argentina	2	7.50	9.13	22
	c.1039-8T_1558?896Tdup	p.520Vfs564X	12–13	Class 5	23	Colombia	2	nd	nd	nd
	c.1105dupT		12	Class 5	Modified from 51	Mexico	1	7.50	7.50	0
	c.1225_1259del	p.Gln409^*^	12	Class 5	UMD	Mexico	1	9.99	9.99	0
	c.1276C > T	p.Gln426^*^	12	Class 5	InSIGHT	Brazil	7	9.99	9.99	0
	c.1333C > T	p.Gln445^*^	12	Class 5	68	Brazil	1	9.99	9.99	0
	c.1360G > C	p.Gly454Arg	12	Class 3	InSIGHT	Uruguay	1	9.99	9.99	0
	c.1459C > T	p.Arg487^*^	13	Class 5	InSIGHT	Brazil	1	11.66	11.66	0
	c.1500_1502delCAT^c,d^	p.Ile501del	13	Class 3	11	Brazil	1	9.15	9.15	0
	c.1558 + 1G > T	p.Val520Glyfs^*^3	13i	Class 5	InSIGHT	Brazil	1	9.15	0.00	−100
	c.1559-2A > C^b^	p.Leu521Lysfs^*^34	13i	Class 4	InSIGHT	Chile	1	10.44	0.00	−100
	c.1559-?_1731 +?del	p.Val520Glyfs^*^7	14–15	Class 5	31	Chile	1	nd	nd	nd
	c.1639_1643dupTTATA	p.Leu549Tyrfs^*^44	14	Class 5	InSIGHT	Brazil	1	6.62	6.62	0
	**c.1681dupT**	p.Tyr561Leufs	11	Not reported/Class 5	Current study	Brazil	1	8.17	8.17	0
	c.1690_1693delCTCA	p.Leu564PhefsTer26	15	Class 5	InSIGHT	Brazil	1	8.17	8.17	0
	c.1724G > A	p.Arg575Lys	15	Class 3	32	Argentina	1	11.78	11.78	0
	c.1731 + 3A > T^b^	p.(Ser556Argfs^*^14)	15i	Class 4	20	Chile	1	11.78	5.63	−52
	c.1732-?_1896 +?del	p.Pro579_Glu633del	16–17	Class 5	InSIGHT	Brazil	1	nd	nd	nd
	c.1763 T > C	p.Leu588Pro	16	Class 3	InSIGHT	Chile	1	9.34	9.34	0
	c.1852_1854 delAAG^d^	p.Lys618del	16	Class 5	InSIGHT	Argentina, El Salvador, Mexico	6	3.51	3.51	0
	c.1853delAinsTTCTT	p.Lys618Ilefs^*^4	16	Class 5	26	Brazil	2	3.51	3.51	0
	c.1855delG^d^	p.Ala619Leufs^*^18	16	Class 5	12, 36	Colombia, Puerto Rico	3	3.51	3.51	0
	**c.1863delG**	p.Met621Ilefs	16	Not reported/Class 5	Current study	Brazil	1	3.51	3.51	0
	c.1890dup^c^	p.Asp631^*^	16	Class 3	26	Argentina	1	3.51	3.51	0
	c.1897-?_1989 +?del	p.Glu633_Glu663del	17	Class 5	InSIGHT	Brazil	1	nd	nd	nd
	c.1897-?_2271 +?del		17–19	Class 5	InSIGHT	Brazil	4	nd	nd	nd
	c.1918C > T	p.Pro640Ser	17	Class 3	InSIGHT	Colombia	1	6.53	6.53	0
	c.1975C > T	p.Arg659^*^	17	Class 5	InSIGHT	Brazil	2	7.70	7.70	0
	**c.1990–93 C > T**		17i	Not reported	Current study	Argentina	1	5.34	5.34	0
	c.1998G > A	p.Trp666^*^	18	Class 5	11	Brazil	1	5.34	5.34	0
	c.2027 T > C^c^	p.Leu676Pro	18	Class 3	26	Brazil	1	5.34	5.34	0
	c.2041G > A^*^	p.Ala681Thr	18	Class 5	UMD	Chile, Brazil, Colombia	7	5.34	5.34	0
	c.2044_2045del	p.Met682Valfs^*^11	18	Class 5	34, 36	Puerto Rico	2	5.34	5.34	0
	c.2059C > T	p.Arg687Trp	18	Class 5	InSIGHT	Brazil	1	8.68	8.68	0
	c.2093C > G	p.Ser698^*^	18	Class 5	InSIGHT	El Salvador	1	8.68	8.68	0
	c.2092_2093delTC	p.Ser698Argfs^*^5	18	Class 5	20	Chile	1	8.68	8.68	0
	c.2103 + 1G > C		18i	Class 4	InSIGHT	Mexico	1	8.68	0.00	−100
	c.2104-?_(*193_?)del	p.S702_X757del	19	Class 5	31	Chile	2	nd	nd	nd
	c.2224C > T	p.Gln742^*^	19	Class 5	26	Brazil	1	7.82	7.82	0
	c.2252_2253dupAA	p.Val752Lysfs^*^32	19	Class 3	InSIGHT	Brazil	1	7.82	7.82	0
	c.2252_2253delAA	p.Lys751Serfs^*^3	19	Class 5	InSIGHT	Argentina	1	7.82	7.82	0
***MSH2***	**c.** ^*^ **32 G > C**		3’UTR	Not reported	Current study	Argentina	1	6.11	6.11	0
	c.71delA	p.Gln24Argfs*40	1	Class 5	32	Brazil	1	10.07	10.07	0
	**c.96dupC**	p.Thr33Hisfs*49	1	Not reported/Class 5	Current study	Brazil	1	10.07	10.07	0
	**c.112G > T**	p.Asp38Tyr	1	Not reported	Current study	Chile	1	10.07	10.07	0
	c.138C > G	p.His46Gln	1	Class 3	InSIGHT	Uruguay	1	10.07	10.07	0
	c.166G > T	p.Glu56*	1	Class 5	InSIGHT	Argentina	1	10.07	10.07	0
	c.174dupC^d^	p.Lys59Glnfs*23	1	Class 5	32	Brazil	3	10.07	10.07	0
	c.181C > T	p.Gln61*	1	Class 5	13	Uruguay	1	10.07	10.07	0
	c.187delG	p.Val63*	1	Class 5	InSIGHT	Brazil	1	10.07	10.07	0
	c.(?_-68)_211 +?del		1	Class 5	InSIGHT	Argentina	1	nd	nd	nd
	c.(?_-68)_645 +?del		1–3	Class 5	InSIGHT	Puerto Rico	2	nd	nd	nd
	c.(?_-68)_1076 +?del		1–6	Class 5	InSIGHT	Argentina	1	nd	nd	nd
	c.212-?_366 +?del	p.Ala72Phefs*9	2	Class 5	InSIGHT	Chile	1	nd	nd	nd
	c.226C > T	p.Gln76*	2	Class 5	InSIGHT, UMD	Mexico	1	8.51	8.51	0
	c.229_230delAG	p.Ser77Cysfs*4	2	Class 5	InSIGHT	Uruguay	1	8.51	8.51	0
	c.289C > T	p.Gln97*	2	Class 5	InSIGHT	Argentina	1	8.83	8.83	0
	**c.367-168C > T**		2i	Not reported	Current study	Argentina	2	6.25	6.25	0
	c.388_389delCA	p.Gln130Valfs*2	3	Class 5	InSIGHT	Brazil, Argentina	3	6.25	6.25	0
	c.425C > G	p.Ser142*	3	Class 5	InSIGHT	Guatemala	1	6.25	6.25	0
	c.435 T > G	p.Ile145Met	3	Class 3	InSIGHT	Argentina	1	6.25	6.25	0
	c.458C > G	p.Ser153Cys	3	Class 3	37	Brazil	1	6.25	6.25	0
	c.484G > A	p.Gly162Arg	3	Class 5	InSIGHT	Argentina	1	6.25	6.25	0
	c.518 T > G	p.Leu173Arg	3	Class 3	InSIGHT	Brazil	1	9.88	9.88	0
	c.528_529delTG	p.Cys176*	3	Class 5	InSIGHT	Brazil	1	9.88	9.88	0
	c.530_531delAA	p.Glu177Valfs*3	3	Class 5	13	Uruguay	1	9.88	9.88	0
	c.557A > G	p.Asn186Ser	3	Class 3	UMD	Uruguay	1	9.88	9.88	0
	c.596delTG	p.Cys199Leufs*15	3	Class 5	12	Colombia	1	9.88	9.88	0
	**c.638dupT**	p.Leu213fs	3	Not reported	44	Mexico	1	9.88	9.88	0
	**c.645 + 1_645 + 10delins15**		3	Not reported/Class 5	Current study	Brazil	1	9.88	0.00	−100
	c.645 + 791_1076 + 4894del	p.Ile217Glufs*28	4–6	Class 5	InSIGHT	Brazil	1	nd	nd	nd
	**c.711_727del17**	p.Ile237Metfs*13	4	Not reported/Class 5	Current study	Brazil	1	7.79	7.79	0
	c.862C > T	p.Gln288*	5	Class 5	InSIGHT	Brazil	1	10.35	10.35	0
	c.876_877insC		5	Class 5	Modified from 36	Puerto Rico	1	8.59	8.59	0
	c.897 T > G	p.Tyr299*	5	Class 5	31	Chile	1	8.59	8.59	0
	Amplification of exon 5		5	Class 5	37	Brazil	1	nd	nd	nd
	c.905 T > A	p.Leu302*	5	Class 5	InSIGHT	Puerto Rico	1	8.59	8.59	0
	**c.914_923delCAGCAGTCAG**	p.Ala305Glufs*23	5	Not reported/Class 5	Current study	Argentina	1	8.59	8.59	0
	c.942 + 3A > T	p.Val265_Gln314del	5i	Class 5	InSIGHT	Brazil	2	8.59	2.54	−70
	c.943-1G > T		5i	Class 5	Modified from 36	Puerto Rico	2	9.59	0	−100
	c.1046C > G	p.Pro349Arg	6	Class 5	InSIGHT	Argentina	1	9.81	9.81	0
	**c.1076 + 1_1076 + 2delGT**		6i	Not reported/Class 5	Current study	Brazil	1	9.81	0.00	−100
	c.1077-?_1276 +?del	p.Leu360Lysfs*16	7	Class 5	InSiGHT	Argentina; Uruguay; Brazil	3	nd	nd	nd
	c.1077-135_1276 + 119dup		7	Class 5	InSiGHT	Brazil	1	8.92	8.92	0
	c.1143_1144insA	p.Arg382Thrfs*7	7	Class 5	37	Brazil	1	5.25	5.25	0
	c.1147C > T	p.Arg382*	7	Class 5	InSIGHT	Brazil	1	5.25	5.25	0
	c.1165C > T	p.Arg389*	7	Class 5	InSIGHT	Colombia	1	5.25	5.25	0
	c.1215C > A	p.Tyr405*	7	Class 5	InSIGHT	Chile	1	8.92	8.92	0
	c.1216C > T	p.Arg406*	7	Class 5	InSIGHT	Uruguay	1	8.92	8.92	0
	**c.1224 T > A**	p.Tyr408*	7	Not reported/Class 5	InSIGHT	Argentina	1	8.92	8.92	0
	c.1226_1227delAG	p.Gln409Argfs*7	7	Class 5	InSIGHT	Brazil	1	8.92	8.92	0
	c.1249delG	p.Val417Leufs*21	7	Class 5	InSIGHT	Brazil	1	8.92	8.92	0
	c.1255C > T	p.Gln419*	7	Class 5	InSIGHT	Brazil	1	8.92	8.92	0
	c.1277-?_1386 +?del	p.Lys427Glyfs*4	8	Class 5	InSiGHT	Brazil	1	nd	nd	nd
	c.1308dupT		8	Class 5	Modified from 36	Puerto Rico	1	10.12	10.12	0
	c.1444A > T	p.Arg482*	9	Class 5	26	Brazil	2	11.59	11.59	0
	c.1447G > T	p.Glu483*	9	Class 5	InSIGHT	Brazil	1	11.59	11.59	0
	c.1457_1460del	p.Asn486Thrfsx10	9	Class 5	InSIGHT	Puerto Rico	1	8.85	8.85	0
	c.1662-2A > G		10i	Class 4	UMD, InSIGHT	Argentina	1	8.01	0.00	−100
	c.1667delT	p. Leu556*	11	Class 5	26	Brazil	1	8.01	8.01	0
	c.1667_1668insA	p.Thr557Aspfs*5	11	Class 5	11	Brazil	1	8.01	8.01	0
	c.1705_1706delGA	p.Glu569Ilefs*2	11	Class 5	InSIGHT	Brazil, Puerto Rico	2	8.01	8.01	0
	c.1738G > T	p.Glu580*	11	Class 5	InSIGHT	Brazil	1	7.82	7.82	0
	c.1759 + 1G > A		11i	Class 4	InSIGHT	Puerto Rico	1	7.82	0.00	−100
	**c.1759 + 57G > T**		11i	Not reported	Current study	Argentina	1	7.82	7.82	0
	c.1777C > T	p.Gln593*	12	Class 5	InSIGHT, UMD	Mexico	1	9.05	9.05	0
	c.1786_1788delAAT	p.Asn596del	12	Class 5	InSIGHT	Brazil	1	9.05	9.05	0
	c.1861C > T	p.Arg621*	12	Class 5	InSIGHT, UMD	Argentina, Brazil	2	9.05	9.05	0
	c.1864C > A	p.Pro622Thr	12	Class 3	14	Argentina	1	9.05	9.05	0
	**c.1865C > G**	p.Pro622Arg	12	Not reported	InSIGHT	Argentina	1	9.05	9.05	0
	c.1911delC^d^	p.Arg638Glyfs*47	12	Class 5	17	Argentina	1	4.78	4.78	0
	c.1967_1970dupACTT	p.Phe657Leufs*3	12	Class 5	26	Brazil	1	4.78	4.78	0
	c.2038C > T	p.Arg680*	13	Class 5	InSIGHT	Chile	1	8.23	8.23	0
	c.2046_2047delTG	p.Val684AspfsX14	13	Class 5	InSIGHT	Argentina	1	8.23	8.23	0
	**c.2078G > A**	p.Cys693Tyr	13	Not reported	Current study	Brazil	1	8.23	8.23	0
	c.2131C > T	p.Arg711*	13	Class 5	InSIGHT	Argentina, Brazil, Chile	4	10.86	10.86	0
	c.2145del	p.Asp716Thrfs*4	13	Class 5	37	Brazil	1	10.86	10.86	0
	c.2152C > T	p.Gln718*	13	Class 5	InSIGHT	Brazil	9	10.86	10.86	0
	c.2178_2179insA		13	Class 5	Modified from 51	Mexico	1	10.86	10.86	0
	c.2185_2192del7insCCCT	p.M729_E731delinsP729_X730	13	Class 5	20	Chile	1	10.86	10.86	0
	c.2187G > T	p.Met729Ile	13	Class 3	26	Brazil	1	10.86	10.86	0
	c.2211-?_2458 +?del	p.Ser738Cysfs*3	14	Class 5	InSIGHT	Brazil	1	nd	nd	nd
	c.2525_2526delAG	p.Glu842Valfs*3	15	Class 5	26	Brazil	2	9.97	9.97	0
	c.2785C > T	p.Arg929*	16	Class 5	26	Brazil, Uruguay	2	6.11	6.11	0
***EPCAM-MSH2***	*EPCAM-MSH2* (exon1–4) deletion		1–4	Class 5	37	Brazil	1	nd	nd	nd
	**c.583C > G**	p.Leu195Val	6	Not reported	Current study	Uruguay	1	nd	nd	nd
	c.555 + 402_*1220del		6–9	Not reported/Class 5	LOVD	Chile	1	nd	nd	nd
	EPCAM:c.(?_1)_(945_?)_MSH2:c.(?_1)_(1076_?)		1–6	Class 5	31	Chile	1	nd	nd	nd
***MSH6***	**c.23_26delACAG**	p.Tyr8SerfsTer8	1	Not reported/Class 5	Current study	Brazil	1	7.38	7.38	0
	c.44C > T	p.Pro15Leu	1	Class 3	ClinVar	Uruguay	1	7.38	7.38	0
	c.124C > T	p.Pro42Leu	1	Class 3	37	Brazil	1	7.38	7.38	0
	**c.457 + 32del**		2i	Not reported	Current study	Argentina	1	10.77	10.77	0
	c.458-?_3172del	p.Gly153_Leu1057del	3–4	Class 5	32	Uruguay	1	nd	nd	nd
	c.663A > C	p.Glu221Asp	4	Class 3	InSIGHT	Uruguay; Argentina	2	10.87	10.87	0
	c.733A > T^*^	p.Ile245Leu	4	Conflicting interpretations of pathogenicity	UMD, Insight	Uruguay	1	10.87	10.87	0
	**c.1133_1134delGA**	p.Arg378Lysfs*3	4	Not reported/Class 5	Current study	Brazil	1	10.87	10.87	0
	c.1338A > T	p.Glu446Asp	4	Class 3	37	Brazil	1	10.87	10.87	0
	c.1483C > T	p.Arg495*	4	Class 5	InSIGHT	Brazil	1	10.87	10.87	0
	c.1519dupA	p.Arg507Lysfs*9	4	Class 5	UMD	Brazil	2	10.87	10.87	0
	c.1591C > A	p.Pro531Thr	4	Class 3	UMD, ClinVAr	Uruguay	1	10.87	10.87	0
	**c.1913delInsAGA**	p.Leu638GlnfsX11	4	Not reported/Class 5	Current study	Brazil	1	8.91	8.91	0
	c.1932G > C	p.Arg644Ser	4	Class 3	37	Brazil	1	8.91	8.91	0
	c.2194C > T	p.Arg732*	4	Class 5	InSIGHT	Brazil	1	8.91	8.91	0
	c.2308_2312delinsT	p.Gly770Cysfs*4	4	Class 5	UMD	Uruguay	1	8.91	8.91	0
	**c.2332_2335dupCTTT**	p.Cys779Serfs	4	Not reported/Class 5	Current study	Brazil	1	8.91	8.91	0
	c.2379_2380delTG	p.Ala794Hisfs*9	4	Class 5	37	Brazil	1	nd	nd	nd
	**c.2659delC**	p.Lys888Serfs*18	4	Not reported/Class 5	Current study	Brazil	1	8.91	8.91	0
	c.2983G > T	p.Glu995*	4	Class 5	InSIGHT	Brazil	1	8.91	8.91	0
	**c.3023C > T**	p.Thr1008Ile	4	Not reported	Current study	Argentina	1	8.91	8.91	0
	c.3119_3120delTT	p.Phe1040*	4	Class 5	InSIGHT	Puerto Rico	1	8.91	8.91	0
	c.3487G > T	p.Glu1163*	6	Class 5	37	Brazil	1	10.55	10.55	0
	**c.3557-144G > A**		6i	Not reported	Current study	Argentina	8	10.29	10.29	0
	**c.3557-185C > T**		6i	Not reported	Current study	Argentina	1	10.29	10.29	0
	c.3632 T > C	p.Leu1211Pro	7	Class 5	InSIGHT	Brazil	1	9.14	9.14	0
	**c.3646 + 91 T > C**		6	Not reported	Current study	Argentina	1	nd	nd	nd
	**c.3772C > G**	p.Gln1258Glu	8	Not reported	Current study	Brazil	1	8.35	8.35	0
	c.3974_3976delAGA	p.K1325del	9	Class 3	37	Brazil	1	6.25	6.25	0
	c.4071ins4		10	Class 3	51	Mexico	1	nd	nd	nd
***PMS2***	c.23 + 72C > T		1i	Class 3	InSIGHT	Argentina	5	8.70	8.70	0
	**c.537 + 187A > G**		5i	Not reported	Current study	Argentina	4	8.04	8.04	0
	c.697C > T	p.Gln233*	6	Class 5	InSIGHT	Brazil	1	6.13	6.13	0
	**c.804-1G > T**		8i	Not reported/Class 5	Current study	Colombia	1	3.54	0.00	−100
	**c.903 + 84 C > T**		8i	Not reported	Current study	Argentina	1	7.64	7.64	0
	**c.903 + 100 T > G**		8i	Not reported	Current study	Argentina	1	7.64	7.64	0
	**c.903 + 144G > T**		8i	Not reported	Current study	Argentina	4	7.64	7.64	0
	c.1004A > G	p.Asn335Ser	10	Class 3	ClinVar	Uruguay	1	10.00	10.00	0
	c.1144G > C	p.Gly382Arg	10	Class 3	37	Brazil	1	10.57	7.69	−27
	c.1211C > G	p.Pro404Arg	11	Class 3	37	Brazil	1	7.77	7.77	0
	c.1239dup	p.Asp414Argfs*44	11	Class 5	37	Brazil	1	7.77	7.77	0
	c.1437C > G	p.His479Gln	11	Class 3	InSIGHT	Argentina	1	7.77	7.77	0
	c.1831dupA	p.Ile611Asnfs*2	11	Class 5	InSIGHT	Argentina	1	9.06	9.06	0
	c.2016delG	p.Met672Ilefs*16	12	Class 5	31	Chile	1	8.61	8.61	0
	c.2036 T > C	p.Ile679Thr	12	Class 3	37	Brazil	1	nd	nd	nd
	c.2182_2184delinsG	p.Thr728Alafs*7	13	Class 5	HGMD	Brazil	2	nd	nd	nd
	**c.2192_2196delTAACT**	p.Leu731Cysfs*3	13	Class 5	InSIGHT	Brazil	1	10.75	10.75	0
	c.2264 T > C	p.Ile755Thr	13	Class 3	37	Brazil	1	nd	nd	nd
	c.2276-?_2445 +?del	p.Ala759Glyfs*8	14	Class 5	InSIGHT	Chile	1	nd	nd	nd

LS: Lynch syndrome; Novel MMR variants are represented in bold; ^a:^ reported as Class 2 by UMD but not assessed by the InSIGHT; ^b^: MMR variant downgraded from Class 5 to Class 4; ^c^: MMR variant downgraded from Class 5 to Class 3; ^d^: MMR variant updated in the nomenclature; nd: not determined

By the MaxEntScan algorithm, we found that 12% of the variants in our cohort are expected to have a negative impact on RNA splicing (Table 3). Indeed, for 27 out of the 220 variants, the MaxEntScan algorithm predicts a significant decrease in splice site strength (>15% decrease in MaxEntScan scores relative to corresponding wild-type splice sites). These include 23 intronic variants (7 within acceptor sites and 16 at donor sites) and 4 exonic variants (located either at the penultimate or at the last position of the exon). Among these variants, 24 are already considered pathogenic (either Class 4 or Class 5, with MaxEntScan scores ranging from −23% to −100% of WT), including 15 variants located at the most conserved positions of the consensus splice sites, i.e. IVS ± 1 or IVS ± 2, and a nonsense mutation located at the penultimate position of *MLH1* exon 8. The three-remaining potential splicing mutations are either currently considered as Class 3 (*MLH1* c.588G + 5G > C, and *PMS2* c.1144G > C) or have not yet been reported (*MLH1* c.588 + 5G > T). Further studies will be necessary to determine if these three variants cause splicing alterations as predicted by MaxEntScan (decrease in donor splice site strength, MaxEntScan scores ranging from −27% to −55% of WT), and if they are pathogenic or not.

Our *in-silico* assessment of potential variant-induced de novo splice sites (data not shown) indicates that 3 out of the 220 variants analyzed in this study are likely to create new splice sites. More precisely, *MLH1* c.117-1G > T is predicted to destroy the acceptor site of *MLH1* exon 2 and to concomitantly create a potential new and stronger acceptor site 5 nucleotides downstream, within the exon; *MSH2* c.645 + 1_645 + 10delins15 is expected to destroy the donor site of *MSH2* exon 3 and to create a new donor site 14 nucleotides downstream the reference site, within intron 8; and *PMS2* c.804-1G > T is predicted to destroy the acceptor site of *PMS2* exon 8 and to concurrently create a new and stronger acceptor site, 8 nucleotides downstream, within the exon. These in silico predictions support the classification of *MLH1* c.117-1G > T, *MSH2* c.645 + 1_645 + 10delins15 and *PMS2* c.804-1G > T as pathogenic (Table 3).

Though the single nucleotide variants (SNV) were spread over the genes, most frequently affected regions included exons 11 of *MLH1* (15%), exon 3 and 7 of *MSH2* (17 and 15%), exon 4 of *MSH6* (65%) and exons 11 and 13 of *PMS2* (31% and 23%).

**Fig. 3:**
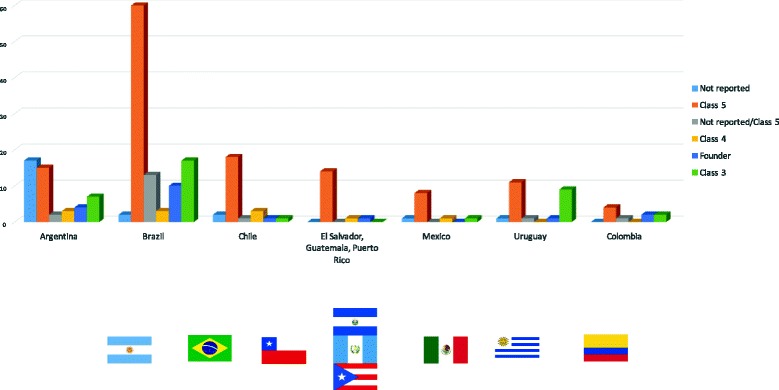
Latin America MMR variants spectrum

We found that the Latin America LS variant spectrum was broad with 80% (175/220) alterations being private i.e., observed in a single family, 15% (33/220) observed in 2–3 families and 6% (12/220) variants observed in ≥4 families. Forty-one variants (19%) had not previously been reported in LS, and thus herein represent novel genetic variants in the MMR genes (including 10 in *MLH1*, 13 in *MSH2*, 11 in *MSH6*, 5 in *PMS2* and 2 in *EPCAM*). The classification of the remaining 179 variants is indicated in Table 3, 37 variants being currently considered as Class 3, 10 as Class 4, 131 as Class 5 and 1 has conflicting interpretations of pathogenicity (Table 3, Fig. 3). The variants have been submitted to the InSiGHT locus-specific database (https://www.insight-group.org).

In total, 45 MMR variants identified in at least two families were classified as recurrent. Among these, the *MLH1* c.1276C > T and the *MSH2* c.2152C > T were identified in ≥7 families from different Brazilian cities and the *MLH1* c.665del was identified in 4 unrelated Uruguayan families. Recurrent pathogenic variants shared by more than one South American country, include: the *MLH1* c.350C > T, c.1852_1854del and the c.2041G > A. More precisely, the *MLH1* c.350C > T was identified in 5 unrelated families from Uruguay and Argentina, the *MLH1* c.1852_1854del was detected in 6 unrelated families from Argentina, Brazil, El Salvador and Mexico, and the *MLH1* c.2041G > A was observed in 7 unrelated families from Chile, Colombia and Brazil. These variants may thus represent frequent *MLH1* variants in South American population. Moreover, we found a high incidence of intronic and not previously reported *MSH6* and *PMS2* variants in Argentina (Table 3).

### Founder variants

Here, we identified 16 international founder variants: 8 in *MLH1,*7 in *MSH2* and 1 in *MSH6* pathogenic variants in 27 LS families [23, 34, 36, 59–74] (Table 4). International founder pathogenic variants detected in >2 unrelated LS families included e.g. *MLH1* c.545 + 3A > G identified as an Italian founder pathogenic variant [75], *MSH2* c.388_389del as a Portuguese founder variant identified in Argentina [69]. The *MSH2* c.942 + 3A > T was found in 2 unrelated Brazilian families and widely described as a Newfoundland founder variant. It had been identified in different populations and could be considered as a world-wide *MSH2* variant [26, 64]. The *MLH1* c.1039-8T_1558 + 896Tdup has been suggested to represent a founder MMR variant in Colombia [23]. In line with the Portuguese influence in Brazil, the *MLH1* c.1897-?_2271 +?del encompassing exons 17 to 19 have been identified in 4 unrelated Brazilian families [69, 70]. The *MLH1* c.2044_2045del have been recently described as a founder variant in Puerto Rico [34, 36] and the *MSH2* c.1077-?_1276 +?del as a Spanish founder Alu-mediated rearrangement which have been identified in Argentina, Uruguay and Brazil [67].

**Table 4:** Founder mutations found in Latin America LS families

Gene	Founder mutation	Total number of LS families (references)	Origin (comments)
*MLH1*	c.306 + 5G > A	1 in Brazil [61]	Spain
*MLH1*	c.545 + 3A > G	2 in Brazil [75]	Italy
*MLH1*	c.1039-8T_1558 + 896Tdup	2 in Colombia [23]	(no haplotype studies were performed)
*MLH1*	c.1558 + 1G > T	1 in Brazil [65]	Italy
*MLH1*	c.1732-?_1896 +?del	1 in Brazil [66, 72]	Finland
*MLH1*	c.1897-?_2271 +?del	4 in Brazil [70, 68]	Portugal (mutation with an estimated age of 283 years)
*MLH1*	c.2044_2045del	2 in Puerto Rico [34, 36]	Puerto Rico
*MLH1*	c.2252_2253delAA	1 in Argentina [40]	Italy (Northern region)
*MSH2*	c.(?_-68)_1076 +?del	1 in Argentina[63, 71, 73]	Italy and North America
*MSH2*	c.388_389del	2 in Argentina and 1 in Brazil [69]	Portugal
*MSH2*	c.942 + 3A > T	2 in Brazil [64]	Newfoundland (considered a world-wide *MSH2* variant)
*MSH2*	c.1077-?_1276 +?del	1 in Argentina, 1 in Uruguay and 1 in Brazil [67]	Spain (Alu-mediated rearrangements)
*MSH2*	c.1165C > T	1 in Colombia [62]	French Canada
*MSH2*	c.1277-?_1386 +?del	1 in Brazil [60]	Italy (Sardinia)
*MSH2*	c.2185_2192del7insCCCT	1 in Chile [20]	Amerindian
*MSH6*	c.2983G > T	1 in Brazil [74]	Finland

*LS* Lynch syndrome

### Update of the MMR variants from the previous South America LS study

Due to changes in InSIGHT classification of variants, 14 variants were altered for the *MLH1* gene and 2 for the *MSH2* gene, relative to our previous classification in Dominguez-Valentin et al. [32]. For *MLH1*, 3 previously classified Class 5 variants were downgraded to Class 4, while 4 previously classified Class 5 were moved to Class 3 and 3 previously classified Class 5 were moved to Class 1 (*MLH1*: c.1558 + 14G > A, c.1852_1853delinsGC, c.1853A > C). Three *MLH1* variants were updated in their nomenclature. For *MSH2* gene, two variants were updated in their nomenclature (Table 3).

### *Differences between LS patients according to the* path*_MMR gene*

The clinicopathological characteristics evaluated were similar between *path*_*MLH1, path*_*MSH2, path*_*MSH6*, *path*_*PMS2* and *path*_*EPCAM* carriers, except for the mean age at CRC diagnosis for *MLH1* (39.6 years) and *MSH2* carriers (41.5 years) (*p* ≤ 0.05) (Table 5). For *path*_*MLH1* carriers, we observed that the probands had more family history of CRC (56.4%) than LS-associated cancers (20.1%) and 97% fulfilled the AMSII criteria. LS individuals with *path_MSH2*, *path_MSH6* and *path_PMS2* were mostly females (63.5%, 90% and 77.8% respectively). *Path*_*MSH2* carriers fulfilled AMSII criteria (100%) while *path_MSH6* and *path_PMS2* carriers had more family history of CRC (30% and 75%, respectively) than LS-associated cancers (10% and 25%, respectively). *Path*_*EPCAM* carriers had a lower number for each clinical characteristic (Table 5). Deviating distributions of the parameters discussed above for *path_MSH6* and especially *path_PMS2* carriers may have escaped significance due to limited number of carriers included.

**Table 5:** Clinicopathologic characterization of LS patients acording to the affected MMR gene

Clinical characteristics	Path_MMR carriers					*p* value
	*Path_MLH1*	*Path_MSH2*	*Path_MSH6*	*Path_PMS2*	*Path_EPCAM*	
Age at CRC diagnosis (mean)*	37.5–41.7 (39.6)*	38.6–41.7 (41.5)*	31.2–43.9 (37.5)	38–58 (48)	38–65 (51.5)	
Gender (n(%))						
Female	39 (54.2)	40 (63.5)	9 (90)	7 (77.8)	1 (33.3)	
Male	33 (45.8)	23 (36.5)	1 (10)	2 (22.2)	2 (66.7)	0.261
Family history of CRC (n(%))						
Yes	53 (56.4)	35 (48.6)	3 (30)	3 (75)	2 (66.7)	
No	41 (43.6)	37 (51.4)	7 (70)	1 (25)	1 (33.3)	0.449
Family history LS associated cancers (n(%))						
Yes	27 (20.1)	18 (25)	1 (10)	1 (25)	2 (66.7)	
No	107 (79.9)	54 (75)	9 (90)	3 (75)	1 (33.3)	0.135
AMSII/Bethesda criteria (n(%))						
AMSII criteria	131(97)	72(100)	8 (100)	2 (66.7)	2 (66.7)	
Bethesda	4 (3)	0	0	0	1 (33.3)	na
Other criteria	0	0	0	1 (33.3)		

*P ≤ 0.05; LS: Lynch syndrome; CRC: colorectal cancer; na: not applied; Path_MMR: Pathogenic (disease-causing) variant of an MMR gene; path_MLH1: pathogenic variant of the MLH1 gene; path_MSH2: pathogenic variant of the MSH2 gene; path_MSH6: pathogenic variant of the MSH6 gene; path_PMS2: pathogenic variant of the PMS2 gene; path_EPCAM: pathogenic variant of the EPCAM gene

The analysis was performed based on available information from Hospital de las Fuerzas Armadas, Uruguay (except for the gender); Clinicas Las Condes, Chile; Hospital Italiano, Argentina; Hospital Espanol de Rosario, Argentina; Hospital de Clinicas, Brazil (except for family history of LS associated cancers) and Clinica del Country, Colombia

### Tumor testing results

Tumors specimens from 83 individuals from Peru, 6 from Argentina, 61 from Bolivia, and 60 from Mexico were analyzed either by IHC and MSI-testing, MSI-testing only, or IHC only, respectively, (Table 6). Of these, 69 (32.8%) were found to have MMR-deficient tumors as determined by IHC or MSI analysis (Table 6). The range of the mean age at diagnosis was 27–43 years for CRC and 37–52 years for endometrial cancer in the different registries. The prevalence of deficient MMR protein expression (MLH1, MSH2, MSH6, PMS2) among Peruvian, Argentinean and Mexican patients was 48%, 50% and 38%, respectively, with most cases having absence of MLH1 protein (data available upon request). Regardless of their MMR proficiency status (proficient vs. deficient), patients had similar ages at CRC diagnosis and gender (Table 7). As shown in Table 7, family history of CRC was increased in MMR-deficient individuals compared to MMR proficient (*P* ≤ 0.05). Interestingly, AMSII criteria were more frequently fulfilled among MMR deficient (42.4%) than MMR-proficient (10.9%) individuals and this difference was statistically significant (*P* ≤ 0.05) (Table 7).

**Table 6:** Summary of hereditary cancer registries data from tumor MMR analysis from suspected Latin America LS families

Latin American Institutions	Number of families	Number of individuals	Age at CRC diagnosis (mean ± SD)	Age at endometrial cancer diagnosis (mean ± SD)	Clinical criteria		MMR deficient (%)	MMR non-deficient (%)
					AMSII	Revised Bethesda		
Instituto Nacional de Enfermedades Neoplásicas (Lima, Peru)^a^	82	83	41(13.1)	52(9.01)	22	60	40(48.2)	43(51.8)
Centro de EnfermedadesNeoplasicas Oncovida (La Paz, Boliva)^b^	46	61	27.7(12.7)	na	46	0	3(4.9)	58(95.1)
Instituto Nacional de Cancerología de México (Mexico City, Mexico)^c^	23	60	33(14.6)	37.5(12.02)	11	12	23(38.3)	37(61.7)
Hospital Privado Universitario de Cordoba (Cordoba, Argentina)^c^	6	6	43.3(8.7)	NA	0	6	3(50.0)	3(50.0)
Total	157	210			79	78	69(32.8)	141(67.2)

a: MMR deficiency analyzed based on IHC and/or MSI; b: MMR deficiency based on BAT-25 MSI marker; c: MMR deficiency based on IHC; NA: not applied; MMR: mismatch-repair; CRC: colorectal cancer; SD: standard deviation; IHC: immunohistochemistry; MSI: microsatellite instability; MSI-H: MSI-high; MSS: microsatellite stable

**Table 7:** Comparison of MMR- deficient versus MMR- proficient individuals from suspected Latin America LS families

Clinical characteristics	MMR status		*p* value
	Deficient	Proficient	
Age at CRC diagnosis (mean + − SD)	42.47	36.3	
Gender (n(%))			
Male	27 (39.1)	36 (34.6)	
Female	42 (60.9)	68 (65.4)	0.545637
Family history of CRC (n(%))			
Yes	66 (98.5)	40 (87)	
No	1 (1.5)	6 (13)	**0.012333**
Family history Lynch syndrome associated cancers (n(%))			
Yes	14 (20.9)	6 (13)	
No	53 (79.1)	40 (87)	0.282626
AMSII/Bethesda criteria (n(%))			
AMSII	28 (42.4)	5 (10.9)	
Bethesda	38 (57.6)	41 (89.1)	**0.000314**

*P ≤ 0.05; *CRC* colorectal cancer, *MMR* mismatch repair

Compilation of IHC and MSI data from reports on Latin America LS cases (published results and/or database entries) revealed that 21% had MMR deficiency based on IHC and/or MSI analysis (2.5%–60%). No information was available for the mean age at CRC and endometrial cancer diagnosis (Table 8). This data highlights the importance of genetic testing for LS in these populations.

**Table 8:** Summary of published data from tumor MMR analysis from suspected Latin America LS families

Latin America published data	Number of families	Number of individuals	Clinical criteria			MMR deficient (%)	MMR non-deficient (%)	Loss IHC					MSI	
			AMSII	Revised Bethesda	Other criteria			MLH1 (%)	PMS2 (%)	MSH2 (%)	MSH6 (%)	PMS1 or MSH3* (%)	MSI-H (%)	MSS (%)
Medellin, Colombia [16]	41	41	4	27	10	14 (34.1)	27 (65.9)	na	na	na	na	na	14 (34.1)	27 (65.9)
Rosario Santa Fe, Argentina [14]	1	3	1	na	na	1 (33.3)	2 (66.7)	na	na	na	na	na	1 (33.3)	2 (66.7)
Sao Paulo, Brazil [15]	106	106	na	na	na	14 (13.2)	92 (86.8)	na	na	na	na	na	14 (13.2)	91 (85.9)
Buenos Aires, Argentina [18]	41	40	16	0	25	18 (45)	22 (55)	12 (30)	na	7 (17.5)	na	na	13 (32.5)	17 (42.5)
Minas Gerais, Brazil [22]	66	66	8	15	43	15 (22.7)	51 (77.3)	na	na	na	na	na	15 (22.7)	51 (77.3)
San Juan, Puerto Rico [21]	164	164	na	na	na	7 (4.3)	157 (95.7)	1 (0.06)	na	6 (3.7)	na	na	1 (0.6)	na
Lima, Peru [24]	90	90	na	na	na	35 (38.9)	55 (61.1)	23 (25.6)	18 (20)	4 (4.4)	2 (2.2)	na	26 (28.9)	64 (71.1)
Rio Grande do Sul, Brazil [25]	212	197	22	100	0	42 (21.3)	155 (78.7)	na	na	na	na	na	42 (21.4)	155 (78.7)
Mexico City, Mexico [27]	10	6	0	5	1	2 (33.3)	4 (66.7)	2 (33.3)	na	0	na	na	na	na
Minas Gerais, Brazil [28]	77	77	10	17	10	16 (20.8)	61 (79.2)	na	na	na	na	na	16 (20.8)	61 (79.2)
Santiago, Chile [31]	35	35	19	16	na	21 (60)	14 (40)	21 (60)	0	6 (17.1)	4 (11.4)	na	28 (80)	7 (20)
Lambayeque, Peru [35]	5	3	5	0	na	1 (33.3)	2 (66.7)	1 (33.3)	1 (33.3)	0	0	na	1 (33.3)	0
Sao Paulo, Brazil [37]	118	118	9	52	57	3 (2.5)	115 (97.5)	3 (2.5)	3 (2.5)	5 (4.2)	5 (4.2)	na	12 (10.2)	na
Santo Andre, SP, Brazil [43]	48	48	2	na	17	13 (27.1)	35 (72.9)	2 (4.2)	3 (6.3)	0	2 (4.2)	9 (19)	na	na
Lima, Peru [45]	28	28	0	0	28	11 (39.3)	17 (60.7)	na	na	na	na	na	11 (39.3)	17 (60.7)
Total	1042	1022	96	232	191	213 (20.8)	809 (79.2)	65 (46.4)	25 (17.9)	28 (20)	13 (9.3)	9 (6.4)	168 (36.9)	287 (63.1)

*MMR* mismatch repair, *MSI* microsatellite instabily, *MSI-H* MSI-high, *MSS* microsatellite stable; *na* not applied, *SD* standard deviation, *IHC* immunohistochemistry

### Family history

Since there are no premonitory signs of susceptibility to LS, family history has been the primary method for identifying patients at risk in Brazil, Mexico, Peru and Paraguay. Four published reports showed that 11.5% (107/931) were selected as likely LS on the basis of a positive family history (Table 9).

**Table 9:** Summary of family history analysis from published data from suspected Latin America LS families

Latin American Databases	Number of families	Number of individuals	Clinical criteria			Interpreted as Sporadic cases	Suspected LS (%)	Non-suspected LS (%)	Median age at CRC diagnosis
			AMSII	Revised Bethesda	Other criteria				
Mexico City, Mexico [49]	210	210	2	0	56	154	2 (0.95)	208 (99.05)	na
Asuncion, Paraguay [50]	324	324	9	0	na	315	9 (2.8)	315 (97.2)	55
Sao Paulo, Brazil [19]	311	311	4	41	213	98	45 (31.5)	266 (85.5)	na
Lima, Peru [33]	86	86	20	31	80	6	51 (59.3)	35 (40.7)	na
*Total*	931	931	35	72	349	573	107 (11.5)	824 (88.6)	

*na* not applied, *MMR* mismatch-repair genes, *CRC* colorectal cancer, *LS* Lynch syndrome

## Discussion

Progress has been achieved throughout the past years regarding a better molecular and clinical characterization of LS in Latin America, which is important for the surveillance and management of high-risk patients and their families [2].

Here, we present the first thorough LS investigation in Latin America by taking into account 15 different countries. We found that germline genetic testing for LS is already available in six of these countries (Argentina, Brazil, Chile, Colombia, Uruguay and Puerto Rico). Moreover, in three countries (Bolivia, Peru and Mexico), where genetic testing is not yet implemented, tumor analyses are already performed for identifying patients most likely to carry a path_MMR variant.

According to our data, the contribution from the different MMR genes is apparently slightly higher for *MLH1* and *MSH2* and lower for *MSH6* and *PMS2* when comparing to the InSIGHT database and international reports. It is possible that this pattern reflects the recent inclusion of *MSH6, PMS2* and *EPCAM* in LS genetic testing in Latin America molecular diagnostic laboratories but could also reflect population structure [32, 48, 76, 77]. Interestingly, the clinicopathological features of path_MMR carriers described in Latin America families are in accordance with other studies, e.g. the AMSII criteria were fulfilled by 64% of the path_MMR carriers [37, 77].

This study revealed that the Latin America spectrum of MMR variants is broad with a total of 220 different variants, of which 80% are currently considered as private, whereas 20% are deemed as recurrent. Our data support evidence on a significant contribution from large deletions/duplications in *EPCAM* and frameshift variants in *MLH1* and *MSH2*. Of the 220 MMR variants, 178 were already listed in the InSiGHT database or previous studies [78, 79], whereas 41 have not been previously reported in LS [80]. In addition, we observed that *MSH2* variants most frequently caused disease in Argentinean LS families. Further studies are needed to elucidate the ancestral origin of MMR variants in this population, which may increase the knowledge on the inheritance of LS among affected Latin America individuals [10, 14, 17, 40].

Differences in the spectrum of path_MMR variants between populations could be due to differences in the sample size, clinical criteria, selection bias, as well as, genetic ancestry of the individual populations. For instance, Caribbean Hispanics have higher percentage of African ancestry compared to Argentineans and Uruguay nationals [36]. Puerto Ricans are an admixed population of three ancestral populations, including European, Africans and Taínos [36]. The South American population is ethnically mixed from American Indian, European, and other ancestries, but the proportions may vary between countries. For instance, European ancestry predominates in Uruguay and Argentina, whereas Brazil includes a more heterogeneous population, which is the result of interethnic crosses between the European colonizers (mainly Portuguese), African slaves, and the autochthonous Amerindians [15]. The Peruvian population is a multi-ethnic population with Amerindian (45%), Mestizo (37%), white Spanish influence (15%), as well as other minority ethnic groups, such as African-American, Japanese, and Chinese (3%) [24]. In Chile, Colombia and Bolivia, Spanish colonist and American Indian ancestry influence the populations [20, 32].

It is well established that awareness of founder variants in a specific geographic area or population can be very helpful in designing cost-effective molecular diagnostic approaches [70, 81, 82]. Founder mutations provide molecular diagnostic centers the benefit of unambiguous results and thereby, do not demand high skilled professional training.

The other aim of the study was to investigate if the previously MMR variants identified in South American LS families [32] are in accordance with the 5-tier classification system [55]. We were able to refine the classification of 16 *MLH1* and *MSH2* variants.

When the tumor MMR data from original and published studies were combined, up to 33% of suspected LS individuals had MMR deficiency. The frequency of MMR deficiency was lower than that reported in studies focusing in American, Spanish and Australian LS families (56%–72%) but is in line to the reported prevalence of MSI in sporadic CRC among Hispanic patients [34, 83–86]. These differences could also be a reflect of the differences in the tumor testing methodologies across the countries, e.g. MSI analysis is not widely available in the majority of routine pathology service laboratories, the number of MSI mononucleotide markers varies between laboratories as well as the limitation in the number of MMR proteins analyzed by IHC. Moreover, even if MMR deficiency is a good predictor of carrying a germline path_MMR variant, MMR deficiency can also result from somatic inactivation, most commonly due to methylation of the *MLH1* promoter [86]. IHC and MSI testing will, however, combined identify most LS patients with high sensitivity and specificity.

In Latin America, low budgets make the issue of integrating genetics into clinical practice a challenge, a situation in which the use of family history becomes important for patient care, as it is a low-cost strategy and a risk assessment tool [19]. In this scenario, published family history data from Paraguay, Peru, Brazil and Mexico suggest its use as a triage tool together with IHC and MSI to identify and stratify genetic risk in these populations [19]. However, awareness of hereditary cancer among clinicians involved in diagnosis and treatment of CRC is currently low, and families actually meeting the clinical criteria may not be identified [77]. In addition, the average life expectancy in Latin America and the Caribbean is 75 years and inequalities persist among and within the countries (www.paho.org). These countries are mainly represented by a young population where family history could be less informative and insensitive for assessing genetic screening for LS.

Limitation on genetic testing has an impact in the evaluation of the patients at risk of hereditary cancer and their relatives, and ultimately increases the burden of cancer for this minority population [35]. As mentioned, in Latin America, genetic testing is not routinely available at the public health system, with exception of few studies conducted in research institutes or private institutions. For instance, until recently the coverage of oncogenetic services in Brazil, was restricted to less than 5% of the population. However, a significant advance took place in 2012, when the coverage of genetic testing by private health care plans became mandatory in Brazil, currently covering around 20–30% of the population [19, 87].

This work provides a snapshot view of the current LS-associated diagnostics practice/output in Latin America. The limitations of this study include the selection of patients recruited from selected reference centers and/or from a nation-wide public reference hospital for cancer patients that cannot renders a representative sample. Furthermore, the diagnostic methodologies may vary between the countries regarding the coverage of the coding region of the genes tested and the clinical criteria for referral to genetic counseling and testing, thus causing an even larger knowledge gap. Finally, several countries are not represented; for instance, we could not find any reports from Venezuela, Honduras, Nicaragua or Ecuador. It will be important to pursue additional studies on LS in Latin America countries to both increase the knowledge of MMR variants in different populations and to bring additional awareness of this condition to medical professionals and public health leaders in Latin America.

## Conclusions

The Latin America LS MMR variants spectrum included new MMR variants, genetic frequent regions and potential founder effect. The present study provides support to set or improve LS genetic testing in these countries. Improving the accessibility, including tertiary care, is vital in low-income and middle-income countries that face an increasing burden of CRC. An early diagnosis and intensive screening may predict the disease and/or improve the disease prognosis. Low cost approaches to reach these ends are discussed.

## Abbreviations

AMS: Amsterdam
AMSII: Amsterdam II criteria
CRC: colorectal cancer
HGMD: Human Gene Mutation Database
HGVS: Human Genome Variation Society
IHC: immunohistochemical
InSIGHT: International Society of Gastrointestinal Hereditary Tumors
LOVD: Leiden Open Variation Database
LS: Lynch syndrome
MMR: mismatch-repair gene
MSI: microsatellite instability
MSI-H: MSI high
MSI-L: MSI low
MSS: microsatellite stable
*path*_*MLH1*: Pathogenic (disease-causing) variant of the *MLH1* gene
Path_MMR: Pathogenic (disease-causing) variant of an MMR gene.
*path*_*MSH2*: Pathogenic (disease-causing) variant of the *MSH2* gene
*path*_*MSH6*: Pathogenic (disease-causing) variant of the *MSH6* gene
*path*_*PMS2*: Pathogenic (disease-causing) variant of the *PMS2* gene
UMD: Universal Mutation Database
